# Clinical characteristics and prognostic factors in patients with breast cancer and leptomeningeal metastases from a large registry of BMBC

**DOI:** 10.1016/j.breast.2025.104433

**Published:** 2025-03-28

**Authors:** Elena Laakmann, Marcus Schmidt, Kristina Lübbe, Elisa Agostinetto, Mette van Ramshorst, Thomas Decker, Wolfram Malter, Francesco Schettini, Mario Fontes Sousa, Carsten Denkert, Tanja Neunhöffer, Leonor Matos, Sabine Linn, Marc Thill, Rudolf Weide, Amanda Fitzpatrick, Marta Vaz Batista, Christoph Mundhenke, Tjoung-Won Park-Simon, Fanny Le Du, Kerstin Riecke, Tanja Fehm, Isabel Witzel, Julia Rey, Valentina Nekljudova, Sibylle Loibl, Volkmar Müller

**Affiliations:** a University Medical Center Hamburg-Eppendorf, Department of Gynecology, Hamburg, Germany; b University Medical Center of the Johannes Gutenberg University Mainz, Department of Gynecology, Mainz, Germany; c Diakovere Henriettenstift, Breast Center, Hannover, Germany; d Université libre de Bruxelles (ULB), Hôpital Universitaire de Bruxelles (H.U.B), Institut Jules Bordet, Medical Oncology Department, Rue Meylemeersch 90, 1070, Bruxelles, Belgium; e Netherlands Cancer Institute, Amsterdam, the Netherlands; f Onkologie, Hämatologie Ravensburg, Germany; g University Hospital of Cologne, Department of Obstetrics & Gynecology, Cologne, Germany; h Medical Oncology Department, Hospital Clinic of Barcelona, Barcelona, Spain; i Translational Genomics and Targeted Therapies in Solid Tumors, August Pi I Sunyer Biomedical Research Institute (IDIBAPS), Barcelona, Spain; j Hospital CUF Tejo & Hospital S. Francisco Xavier, Lisbon, Portugal; k Institute of Pathology, University Hospital Marburg, Baldingerstraße, 35043, Marburg, Germany; l Frauenärzte am Dom, Mainz, Germany; m HELIOS Dr. Horst Schmidt Kliniken Wiesbaden, Germany; n Breast Cancer Unit, Champalimaud Clinical Centre, Champalimaud Foundation, Lisbon, Portugal; o Department of Gynecology and Gynecological Oncology Agaplesion Markus Krankenhaus, Frankfurt, Germany; p Institut für Versorgungsforschung in der Onkologie, Koblenz, Germany; q Kings College London, London, United Kingdom; r Fernando Fonseca EPE, Amadora, Portugal; s Clinic Bayreuth, Bayreuth, Germany; t University Medical Center Schleswig-Holstein, Kiel, Germany; u Hannover Medical School, Department of Gynecology, Hannover, Germany; v Centre Eugène Marquis, Rennes, France; w Universitätsklinikum Düsseldorf, Germany; x CIO ABCD, Germany; y Department of Gynecology, Universitätsspital Zürich, University of Zurich, Zurich, Switzerland; z German Breast Group (GBG) Forschungs GmbH, Neu-Isenburg, Germany

**Keywords:** Leptomeningeal metastasis, Breast cancer, Registry

## Abstract

**Background:**

Leptomeningeal metastases (LM) in patients with breast cancer (BC) are associated with a dismal prognosis. We explored clinical characteristics and prognostic factors in patients with BC and LM in the German Brain Metastases in Breast Cancer Registry.

**Methods:**

All patients with histologically confirmed BC and diagnosis of LM (defined as the presence of tumor cells in the cerebrospinal fluid, or presence of typical clinical symptoms in combination with typical magnetic resonance imaging findings) were included.

**Results:**

A total of 3857 patients were included in the analysis (n = 859 (22.3 %) with LM). Among patients with LM a median progression-free survival was 4.2 months (95 % CI 3.6–4.8), and median overall survival was 5.7 months (95 % CI 4.9–6.7). In the multivariate analysis older age ( ≥ 60 vs. <60 years, Hazard ratio (HR): 1.65, 95 %CI: 1.25–2.18), worse performance status (ECOG 2–4 vs. 0–1 HR: 2.15, 95 %CI: 1.63–2.82), hormone receptor positive/HER2-negative (HR+/HER2-) or triple-negative subtype (HR: 1.54 95CI%: 1.07–2.23 and HR: 1.87, 95 %CI: 1.25–2.81), and higher number of BM (2–3 vs. 1, HR: 1.49, 95 %CI: 1.05–2.11 4) were significantly associated with a higher risk of death. Stereotactic radiotherapy (HR 0.49 95 %CI 0.30–0.79) and whole brain irradiation (HR: 0.58, 95 %CI: 0.42–0.80), endocrine therapy in patients with HR + BC (HR: 0.31, 95 %CI: 0.21–0.45) as well as HER2-targeted therapy for patients with HER2+ BC (HR 0.41, 95 %CI: 0.25–0.68) were associated with a significantly longer survival.

**Conclusions:**

Clinicopathological factors associated with survival can help clinicians identify patients who are candidates for treatment (de)escalation in clinical trials.

## Highlights

• Analysis of 859 patients with leptomeningeal metastases in the BMBC registry.
• High need to optimize survival outcomes: median progression-free survival 4.2 months, median overall survival 5.7 months.
• Older age, worse performance status, hormone receptor positive/HER2-negative or triple-negative subtype and higher number of BM are significantly associated with a higher risk of death.
• Endocrine therapy in patients with hormone receptor positive breast cancer and HER2-targeted therapy for patients with HER2 positive breast cancer are associated with a significantly longer survival.
• Clinicopathological factors associated with survival can help clinicians identify patients who are candidates for treatment (de)escalation in clinical trials.

**Key points**

- Prognosis of patients with BC and LM is significantly worse in comparison to patients with BC and BM without LM.
- The identified clinicopathological factors can help to identify patients who are candidates for treatment (de)escalation in clinical trials.

**Importance of the study**

Patients with LM have a short survival, which indicates an unmet clinical need for the optimization of the treatment of LM and development of strategies to prevent LM. Analysis of the large cohort of patients from the BMBC registry shows that the prognosis of patients with BC and LM is significantly worse in comparison to patients with BC and BM without LM. The identified prognostic factors for patients with LM can support the clinicians to identify groups of patients with potential for better survival who could possibly benefit from a more intense treatment regime and on the other hand to identify patients with a worse survival for whom a best supportive care concept would be a reasonable option. Also, our data provides a rationale to more often consider HER2-targeted therapy for patients with LM and HER2+ disease, because of its shown favorable effect on survival.

## 1 Introduction

Leptomeningeal metastases (LM) are defined as the spread of tumor cells within the leptomeninges and the subarachnoid space [1].

Up to 5 % of patients with breast cancer (BC) develop LM during the course of the disease. The risk is higher for patients with lobular BC or triple-negative BC (TNBC) [2]. The development of LM is associated with poor prognosis, with a median survival of 4 months [3,4]. Furthermore, the incidence of LM is rising, possibly due to longer survival of patients with metastatic BC and due to more sensitive diagnostic tools [[1], [5], [6]].

Nowadays, limited evidence is available concerning the specific clinical characteristics and optimal therapeutic approach in patients with BC and LM. Most of the published data on LM evaluated mixed cohorts of patients with different primary tumors. Specific factors associated with prognosis of patients with BC and LM have not been characterized in a large patient cohort.

The aims of this retrospective, cohort study were to characterize patients with BC and LM within the German Brain Metastases in Breast Cancer Registry (BMBC), to explore their survival outcomes and to identify potential features to allow for a better prognostic stratification.

## 2 Materials and methods

### 2.1 Patient population/data source

The BMBC registry collects clinical data and tumor samples of patients with brain metastases (BM) and BC [[7], [8], [9], [10]].

Clinical data of over 4000 patients with central nervous system metastases (CNS) and BC from 113 centers in Germany were included in the registry, between 01.01.2014 and 07.01.2023. The project is coordinated by the University Medical Center Hamburg-Eppendorf and the German Breast Group, in collaboration with the German AGO Breast study and TRAFO translational research groups.

In the present analyses, patients included in the BMBC registry before 07.01.2023 (taking a data snapshot from June 4, 2023 after data cleaning) were screened for eligibility. All patients with histologically confirmed BC and diagnosis of LM and/or BM were included (diagnosis of LM was defined as the presence of tumor cells in the cerebrospinal fluid, or presence of typical clinical symptoms in combination with typical magnetic resonance imaging (MRI) findings, as *per* EANO-ESMO criteria [10,11]

### 2.2 Objectives

The main objectives of this retrospective, cohort study were to describe the clinico-pathological characteristics and the treatment modalities of BC patients with LM versus patients without LM (but with BM), and to evaluate and compare their survival outcomes of overall survival (OS) and progression-free survival (PFS). OS was defined as the time from date of diagnosis of BM or LM, whichever occurred first, to the date of death; PFS was defined as the time from the date of first diagnosis of BM or LM to disease progression (either intracranial or extracranial) or death, whichever occurred first.

Further objective was to analyze the PFS and OS in the subgroup of patients with LM adjusting for the following covariates:

- Age at diagnosis of BM
- Hormone receptor (HR)-status
- Eastern Cooperative Oncology Group (ECOG) performance status
- Biological subtype (HR+/HER2+ vs. HR-/HER2+ vs. Luminal A/B-like vs. TNBC)
- Number, maximal size and clinical signs of BM at diagnosis
- Extracranial metastases (ECM) at the diagnosis of BM
- Type of radiotherapy
- Cytologically confirmed LM at diagnosis of BM
- Systemic therapy

In the presented analysis, hormone receptor (HR) and Human Epidermal Growth Factor Receptor 2 (HER2) status were defined according to American Society of Clinical Oncology (ASCO)/College of American Pathologists (CAP) guidelines [12,13].

### 2.3 Statistical analyses

Patient characteristics were summarized using descriptive statistics. Clinical and pathological characteristics of patients with and without LM were compared by Wilcoxon rank-sum test for continuous variables and the Fisher's exact test or Pearson χ^2^-test, whenever appropriate, for categorical variables.

OS and PFS were estimated using Kaplan-Meier method and compared between patients with vs without LM by using the log-rank test. Median survival times and survival rates after 1,2,3 and 4 years were reported.

Univariate and multivariate Cox hazard modes were performed to report hazard ratios (HR) with the corresponding 95 % confidence intervals (CI) and to adjust for covariates.

All reported p-values were two-sided, the significance level was set to 0.05. The data was analyzed using SAS® (Statistical Analysis Software) version 9.4 with SAS Enterprise Guide Version 8.3 on Microsoft Windows 10 Enterprise.

## 3 Results

### 3.1 Characteristics of patients with leptomeningeal metastases

**Fig. 1:**
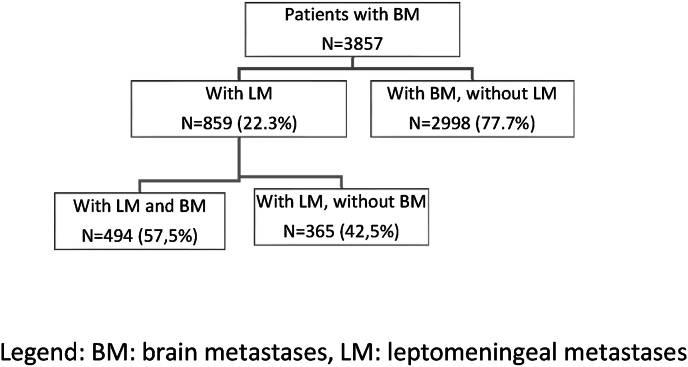
Distribution of patients with and without LM in the BMBC registry. Legend: BM: brain metastases, LM: leptomeningeal metastases.

Clinical data of 3857 patients from the BMBC registry was available for the analysis. Overall, 859 (22.3 %) patients had a diagnosis of LM, of whom 365 (42.5 %) had isolated leptomeningeal disease without BM (Fig. 1). Leptomeningeal disease was cytologically confirmed in 396 (46.1 %) patients. In 463 (53.9 %) patients, the diagnosis of LM was based on clinical symptoms in combination with MRI results and based on the clinical information of patients who were documented to have received intrathecal therapy.

Median time between primary BC diagnosis and disease detection in CNS (BM and/or LM) in the overall cohort was 36.3 months (95 %CI 34.9–38.0); 35.9 months (95 %CI 34.0–37.4) in the cohort without LM and 39.1 (95 %CI 35.7–43.8) months in the cohort with LM.

Among patients with LM, the median age at diagnosis of BC was 51 years (range 24–85 years), while median age at diagnosis of any CNS metastases was 58 years (range 25–86 years). Of 408 patients with available performance status according to the ECOG, 345 (84.6 %) had a good or moderately restricted performance status (i.e. PS of 0–2) at diagnosis of CNS metastases. Of the patients with LM and available primary tumor features 173 (22.2 %) patients had HR+/HER2+ BC, 92 (11.8 %) HR-/HER2+, 349 (44.8 %) HR+/HER2-, and 165 (21.2 %) triple-negative BC (TNBC). An overview of the HER2 IHC Status at timepoint of BC and CNS Metastases is provided in the Supplementary Table 1. The majority of patients (80 %) had distant extracranial metastases at the time of first diagnosis of CNS metastases. Detailed information concerning clinical characteristics of patients with and without LM is shown in Table 1 and supplementary material (Suppl. Table 2).

**Table 1:** Baseline characteristics.

Parameter	Category	Patients without LM N = 2998 N (%)	Patients with LM N = 859 N (%)	Overall N = 3857 N (%)	p-value∗
Age at first diagnosis of BC, years	Median	52.0	51.0	52.0	0.321
	Min, Max	20.0, 98.0	24.0, 85.0	20.0, 98.0	
Age at diagnosis of BM, years	Median	57.0	58.0	57.0	0.771
	Min, Max	22.0, 99.0	25.0, 86.0	22.0, 99.0	
Max diameter of BM, cm	Mean	2.3	2.1	2.2	0.003
ECOG/Karnofsky performance status at diagnosis of BM	ECOG 0 (100 %)	228 (16.7)	53 (13.0)	281 (15.8)	0.010
	ECOG 1 (80–90 %)	621 (45.5)	168 (41.2)	789 (44.5)	
	ECOG 2 (60–70 %)	366 (26.8)	124 (30.4)	490 (27.6)	
	ECOG 3 (40–50 %)	122 (8.9)	45 (11.0)	167 (9.4)	
	ECOG 4 (10–30 %)	29 (2.1)	18 (4.4)	47 (2.6)	
HER2-status at BM	negative	270 (42.1)	70 (43.8)	340 (42.4)	0.721
	positive	372 (57.9)	90 (56.3)	462 (57.6)	
HER2-status switch	no switch	736 (87.9)	186 (87.7)	922 (87.9)	0.907
	switch of HER2-Status from Histology of BC to BM	101 (12.1)	26 (12.3)	127 (12.1)	
Biological subtype of primary BC∗∗ (4 categories)	HR+/HER2+	800 (29.3)	173 (22.2)	973 (27.7)	<.001
	HR-/HER2+	451 (16.5)	92 (11.8)	543 (15.5)	
	HR+/HER2-	849 (31.1)	349 (44.8)	1198 (34.1)	
	TNBC	631 (23.1)	165 (21.2)	796 (22.7)	
Pathological stage (pT)	pT0-T2	1414 (79.6)	413 (78.8)	1827 (79.4)	0.712
	pT3-T4	362 (20.4)	111 (21.2)	473 (20.6)	
	N0	638 (37.1)	162 (32.5)	800 (36.1)	0.064
	N1	1080 (62.9)	336 (67.5)	1416 (63.9)	
Pathological stage (ypN)	ypN0	980 (39.0)	245 (34.3)	1225 (37.9)	0.026
	ypN+	1535 (61.0)	469 (65.7)	2004 (62.1)	
Tumor grading of primary BC	G1	58 (2.2)	10 (1.3)	68 (2.0)	<.001
	G2	1061 (39.7)	368 (47.3)	1429 (41.4)	
	G3	1551 (58.1)	400 (51.4)	1951 (56.6)	
Number of BM	1	852 (29.9)	253 (38.2)	1105 (31.5)	<.001
	2–3	771 (27.1)	160 (24.1)	931 (26.5)	
	≥4	1223 (43.0)	250 (37.7)	1473 (42.0)	
Neurological symptoms at diagnosis of BM	no	704 (23.5)	150 (17.5)	854 (22.1)	<.001
	yes	2294 (76.5)	709 (82.5)	3003 (77.9)	
ECM at diagnosis of BM∗∗∗∗	no	572 (19.1)	170 (19.8)	742 (19.3)	0.659
	yes	2422 (80.9)	689 (80.2)	3111 (80.7)	

Legend: LM leptomeningeal metastases, ECOG Eastern Cooperative Oncology Group, ECM extracranial metastases, BC breast cancer, BM brain metastases.

∗Fisher's exact test resp. Chi-squared test between patients with vs. without LM.

∗∗If HER2-Status at diagnosis of BC was unknown, but Anti-HER2-targeted therapy was given, the subtype was set to HR+/HER2+ resp. HR-/HER2+ (if information about HR-status was given).

∗∗∗ Diagnosis of ECM not later than 60 days after diagnosis of BM.

Patients with LM had a significantly worse ECOG performance status at diagnosis of CNS metastases, compared to patients without LM (ECOG 2–4, 45.8 % vs. 37.8 %, P = 0.010). Concerning the tumor biology, patients with LM had significantly more often HR+/HER2-disease (45.2 vs. 31.8 %, P < 0.001), significantly more often an invasive-lobular tumor biology (13.4 % vs. 6.3 %, P < 0.001), significantly less common HER2+ tumors (34.2 % vs. 46.3 %, P < 0.001) and significantly less common G3 tumor differentiation grade (51.4 vs. 58.1 %, P < 0.001). Furthermore, patients with LM had a significantly lower number of BM (38.2 % vs. 29.9 % with 1 BM, P < 0.001) but a significantly higher rate of neurological symptoms (82.5 vs. 76.5 %, P < 0.001). In those who have received a neoadjuvant chemotherapy, the percentage of patients with positive locoregional lymph nodes after treatment for early BC was significantly higher in patients with LM in comparison to patients without LM in the follow-up (ypN+ 65.7 vs. 61.0 %, P = 0.026). There was no statistically significant difference in the tumor size of the primary BC, nor the presence of extracranial metastases between patients with and without LM. However patients with bone metastases as first site of ECM developed LM more often (Supplementary Material Table 2).

### 3.2 Therapy modalities of patients with LM

After the diagnosis of CNS metastases patients with LM were significantly more often treated with chemotherapy than patients with BM only (39.2 % versus 35.3 %, P = 0.036). Endocrine therapy (in patients with HR + BC) was administered at a similar rate in patients with versus without LM (23.6 % versus 22.6 %, P = 0.654). Concerning further targeted treatment options, CDK4/6 inhibitors were used at a similar rate between those with a HR + LM (5.2 %, 31 of 598 patients) and with a HR + BM (4.4 %, 83 of 1866 patients).

In patients with HER2-positive tumors, HER2-targeted therapy was significantly less common administered in patients with LM compared to patients with BM but absence of LM (34.6 vs. 42.3 %, P = 0.015). An overview of the different applied HER2-targeted treatments after diagnosis of BM is presented in Table 3.

**Table 2:** Distribution of intrathecal therapies in patients who received intrathecal therapies after diagnosis of BM.

Intrathecal (i.t.) therapy	Category	HR+/HER2+ N = 11 N (%)	HR-/HER2+ N = 6 N (%)	Luminal A/B like N = 29 N (%)	TNBC N = 13 N (%)	Overall N = 59^a^ N (%)
Cytarabine i.t.	no	5 (50.0)	4 (80.0)	16 (55.2)	9 (69.2)	37 (61.7)
	yes	5 (50.0)	1 (20.0)	13 (44.8)	4 (30.8)	23 (38.3)
	missing	1	1	0	0	2
Trastuzumab i.t.	no	9 (90.0)	4 (80.0)	29 (100)	13 (100)	58 (96.7)
	yes	1 (10.0)	1 (20.0)	0 (0.0)	0 (0.0)	2 (3.3)
	missing	1	1	0	0	2
MTX i.t.	no	4 (40.0)	2 (40.0)	11 (37.9)	2 (15.4)	19 (31.7)
	yes	6 (60.0)	3 (60.0)	18 (62.1)	11 (84.6)	41 (68.3)
	missing	1	1	0	0	2
Thiotepa i.t.	no	10 (100)	5 (100)	28 (96.6)	13 (100)	59 (98.3)
	yes	0 (0.0)	0 (0.0)	1 (3.4)	0 (0.0)	1 (1.7)
	missing	1	1	0	0	2

The percentages do not sum up to 1. In 10 patients more than one intrathecal therapy was documented (N = 9 with MTX and Cytarabine, N = 1 MTX and Thiotepa).

a According to Statistical Report v3.0 62 patients received an intrathecal therapy, but no information about type of i.t. therapy was given in 3 patients.

**Table 3:** Distribution of systemic HER2-targeted treatment after the diagnosis of BM in patients with HER2-positive BC.

Parameter	Category	Patients without LM N = 1422 N (%)	Patients with LM N = 309 N (%)	Overall HER2+ patients^a^l N = 1731 N (%)
HER2-targeted therapy	yes	601 (42.3)	107 (34.6)	708 (40.9)
Specification of HER2-targeted therapy^a^				
Trastuzumab	yes	334 (55.6)	61 (57.0)	395 (55.8)
Trastuzumab + Pertuzumab	yes	25 (4.2)	1 (0.9)	26 (3.7)
Lapatinib-containing therapy	yes	271 (45.1)	51 (47.7)	322 (45.5)
T-DM1	yes	264 (43.9)	45 (42.1)	309 (43.6)
Tucatinib-containing therapy	yes	33 (5.5)	7 (6.5)	40 (5.6)
Trastuzumab Deruxtecan	yes	27 (4.5)	7 (6.5)	34 (4.8)

Legend. LM: leptomeningeal metastasis.

a HER2-positive patients either at diagnosis of BC, at diagnosis of BM or at diagnosis of ECM.

Sixty-two (7.2 %) patients with LM were treated with intrathecal therapy (Table 2).

3 patients with LM and 19 patients with CNS metastases without LM were treated with an immune checkpoint inhibitor. Sacituzumab govitecan was applied in 3 patients with LM and 7 patients with CNS metastases without LM.

Furthermore, the application of the HER2 targeted therapy was analyzed for two different time periods (before and after 2018). Before 2018 41.4 % of patients (n = 465) without LM were treated with a HER2-targeted therapy vs. 34.5 % (n = 77) with LM (P = 0.062). After 2018 45.5 % (n = 136) patients without LM were treated with a HER2-targeted agent vs. 34.9 % (n = 30) with a LM (P = 0.085).

Among LM patients who also had BM, 38 (6.2 %) had undergone BM resection. 134 (21.9 %) patients received both BM resection and radiotherapy of the brain. A total of 440 patients (71.9 %) were treated with radiotherapy only. The applied radiotherapy type is shown in the Supplementary Table 3.

### 3.3 Survival analysis of patients with versus without leptomeningeal metastases

**Fig. 2:**
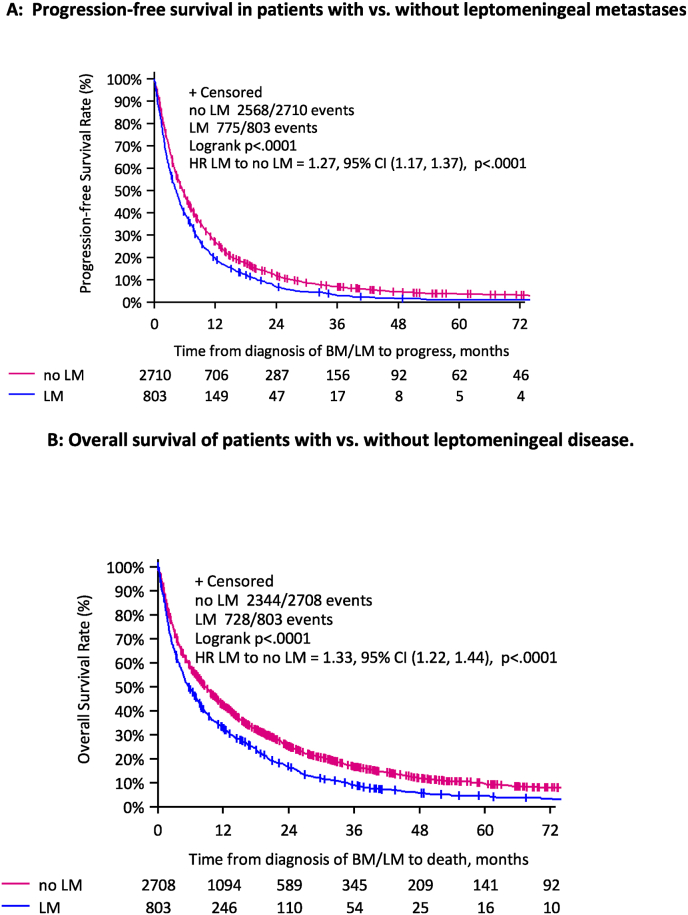
A: Progression-free survival in patients with vs. without leptomeningeal metastases. B: Overall survival of patients with vs. without leptomeningeal disease. Legend. LM: leptomeningeal metastasis; BM: brain metastasis; HR: hazard ratio for progression-free survival; CI: confidence interval.

Median follow-up time in the overall cohort was 19.1 years from the diagnosis of BC and 5.3 years from diagnosis of CNS metastases. Median PFS was 4.2 (95 % CI 3.6–4.8) months in patients with LM and 5.6 (95 % CI 5.2–5.9) months in the cohort of patients without LM (Fig. 2). The difference between the PFS in both cohorts was statistically significant (P < 0.001). Median OS of patients with LM was 5.7 months (95 % CI 4.9–6.7) and was significantly shorter compared to median OS of patients without LM (8.7 months (95 % CI 8.0–9.4; P < 0.0001, Fig. 2 A and 2 B).

The estimated 1-year OS in patients with LM was 32.7 % (95 %CI 29.5–36.0) and 42.4 % (95 %CI 40.5–44.2) among patients without LM. The 4-year survival rate was 5.5 % (95 % CI 3.8–7.5) in patients with LM and 11.8 % (95 %CI 10.5–13.2) in patients without LM (Supplementary Fig. 1).

### 3.4 Survival analysis of patients with leptomeningeal metastases

In univariate analyses, several factors were associated with a significantly worse prognosis in patients with a LM; namely, older age at diagnosis of BC, older age at diagnosis of BM, HR+/HER2-or TNBC subtypes, higher number of BM, worse ECOG, neurological symptoms at time of CNS metastases diagnosis and ECM at time of BM diagnosis. HER2-positivity of the primary breast cancer, radiotherapy of the brain as well as systemic chemotherapy, endocrine therapy for HR+ and HER2-targeted therapy for HER2+ tumors after the diagnosis of CNS metastases were significantly associated with a longer survival (Supplementary Table 4). The estimated OS rates in the cohort of patients with and without cytological confirmation of LM did not differ significantly (Supplementary Fig. 2). The use of an intrathecal therapy did not show a significant correlation with OS. More details are reported in Fig. 2 (supplementary material).

In the multivariate analysis, the following factors were significantly associated with a higher risk of death: age ( ≥ 60 vs. <60 years, HR: 1.65, 95 %CI: 1.25–2.18, P < 0.001), worse performance status at diagnosis of CNS metastases (ECOG 2–4 vs. 0–1 HR: 2.15, 95 %CI: 1.63–2.82), a HR+/HER2-or triple-negative tumor biology vs. triple-positive (HR: 1.54 95CI%: 1.07–2.23, P = 0.02 and HR: 1.87, 95 %CI: 1.25–2.81, P = 0.003, respectively) as well as a higher number of BM (2–3 vs. 1, HR: 1.49, 95 %CI: 1.05–2.11, P = 0.03). The following factors were significantly associated with a longer survival: stereotactic therapy of BM (HR 0.49 95 %CI 0.3–0.79, P = 0.004) and whole brain irradiation (WBRT) (HR: 0.58, 95 %CI: 0.42–0.80, P = 0.001) vs. no radiotherapy of the CNS metastases, endocrine therapy in patients with a HR + BC (HR: 0.31, 95 %CI: 0.21–0.45, P < 0.001) as well as HER2-targeted therapy for patients with a HER2+ BC (HR 0.41, 95 %CI: 0.25–0.68, P < 0.001, Table 4).

**Table 4:** Multivariate Cox Regression of the time from BM/LM to death in patients with LM.

Parameter	Category	Hazard ratio	95 % CI	p-value
Age at BM diagnosis	<60			
	≥60	1.65	(1.25, 2.18)	<.001
ECOG at BM diagnosis	ECOG 0-1			
	ECOG 2-4	2.15	(1.63, 2.82)	<.001
Biological subtype^a^	HR+/HER2+			.004
	HR-/HER2+	.784	(.452, 1.36)	.387
	HR+/HER2-	1.54	(1.07, 2.23)	.021
	TNBC	1.87	(1.25, 2.81)	.003
Number of BM	1			.076
	2–3	1.49	(1.05, 2.11)	.025
	≥4	1.29	(.929, 1.78)	.129
Clinical Symptoms	no			
	yes	1.45	(.969, 2.18)	.070
ECM at BM diagnosis	no			
	yes	1.20	(.827, 1.73)	.340
Radiotherapy	No RTH			.001
	Stereo RT only	.488	(.301, .793)	.004
	WBRT only	.582	(.424, .798)	.001
	WBRT and Stereo RT	.433	(.169, 1.11)	.082
	RTH unknown	2.88	(.637, 13.0)	.169
Chemotherapy after diagnosis of BM	no			
	yes	.765	(.585, 1.00)	.050
Hormone therapy after diagnosis of BM	no			
	yes	.305	(.207, .450)	<.001
Targeted therapy after diagnosis of BM	no			
	yes	.408	(.247, .675)	<.001

Legend. BM: brain metastasis; FU: follow-up; WBRT: whole breast radiotherapy; RT: radiotherapy; HR: hormone receptor; +: positive; -: negative; ECM: extracranial metastases; ER: estrogen receptor; PgR: progesterone receptor; BC: breast cancer.

a If HER2-Status at diagnosis of BC was unknown, but Anti-HER2-targeted therapy was given, the subtype was set to HR+/HER2+ resp. HR-/HER2+ (if information about HR-status was given, too).

### 3.5 Survival analyses in patients with versus without LM according to different time periods of BM diagnosis

Patients with BM without LM experienced a better OS regardless of the time period of diagnosis, as compared to patients with LM. A more pronounced numerical survival benefit in patients with BM without LM, as compared to patients with LM was observed in the decade 2010–2019, as compared to 2000–2009 and 2020–2022. Namely, median OS for BM without LM vs. median OS for LM: 8.1 (95 %CI: 7.2–9.6) vs. 4.7 months (95 %CI: 3.5–7.1) in 2000–2009, 9.1 (95 % 8.3–10.0) vs. 6.2 months (95 %CI: 5.0–7.1) in 2010–2019 and 7.8 (95 %CI: 5.8–9.9) vs. 4.9 months (95 %CI: 3.6–9.2) in 2020–2022.

## 4 Discussion

In this study we analyzed data of patients with BC and BM with or without leptomeningeal involvement. Patients who developed LM had more often HR+/HER2-primary tumors and a histological grade 1–2, suggesting that development of LM may occur independently of the aggressiveness of the primary BC features. Furthermore, we observed no clear correlation with the extent of the extracranial metastatic disease. Further working groups made similar observations [9,10,[14], [15], [16], [17]]. These findings support the hypothesis that LM has a unique tumor biology, as recently shown in genomic studies [18] and is not simply a manifestation of widespread advanced disease.

Translational research projects within the BMBC Registry are planned to better understand the biology of LM.

The survival data of patients with a LM is in line with previous published evidence of poor survival associated with LM, with a median OS of 5.7 months in our LM cohort. No improvement of survival was observed in the recent time period. A slight decrease in survival could be explained by the lower number of patients with LM included in BMBC registry at this time period. The poor survival emphasizes the need to better understand this disease entity and to preferably include these patients in clinical trials to find treatment strategies that may increase survival and preserve quality of life [6,10,11,[19], [20], [21]]. In addition, we showed that HER2-targeted treatment was significantly less often applied in patients with HER2-positive BC and LM compared to patients with BM but without LM, while HER2-targeted therapy was associated with a significant improvement in survival. This information provides a rationale to rethink the clinical routine and to consider HER2-targeted therapy for patients with LM more often in appropriate cases, particularly those which have shown CNS activity (trastuzumab deruxtecan and tucatinib). The relatively low rate of HER2-targeted treatment in our cohort reflects the real-world situation regarding the use of targeted agents in patients with CNS metastases.

Unfortunately, only a very small proportion of patients in this registry was treated with novel HER2-targeted agents like antibody-drug conjugates or tyrosine kinase inhibitors, which are extremely active drugs, also effective in central nervous system level [[22], [23], [24]]. Whether these and other promising new drugs might be associated with improved outcomes in patients with LM should be a priority area of investigation, considering the dismal prognosis of this patient subgroup and the potential of these drugs to be a gamechanger in this scenario [25]. A possible explanation of a relatively low rate of the application of (modern) HER2-targeted agents in patients with a LM in our registry is that the majority of the patients were included in the retrospective part of the registry.

We could not identify a better survival in patients with intrathecal therapy, but the number of patients treated with intrathecal therapy was maybe too low to find a significant association. The use of intrathecal treatment in addition to systemic treatment versus systemic treatment alone was tested in several clinical trials, which showed heterogeneous results [10,11,26,27]. This topic should be further evaluated in randomized prospective trials.

Furthermore, we could identify a patient cohort with LM with a better prognosis, i.e. patients at younger age, better performance status, triple-positive tumor biology, low number of BM as well as patients receiving endocrine therapy (if HR + tumor biology), HER2-targeted therapy (if HER2+ tumor) or radiotherapy of the brain. Prospective interventional studies are urgently needed to evaluate if this patient cohort might gain benefit from a more intense therapeutic approach. The better survival of patients who received whole brain radiotherapy must be interpreted with caution. Whole brain irradiation was commonly used in patients treated for several years. In contrast, patients with a poor prognosis were commonly advised for a best supportive care concept. Furthermore a possible benefit of a stereotactic radiotherapy could be an effect of limited CNS disease.

It is important to acknowledge the limitations of this study, primarily related to its retrospective nature. All data were extracted from multiple centers in Germany, each with its own standards of care. Over the course of the study period, advancements in BC treatment have been made, and may have influenced patient clinical outcomes. This is especially relevant for the interpretation of survival data, that should be considered with caution.

However, our study is the first to include such a uniquely large number of patients with BC-specific BM and LM, providing insights on clinicopathological features and outcomes, as well as hints suggesting the role of a diverse range of treatment modalities.

Overall, an especially unfavorable outcome for patients with LM, as compared to patients with BM without LM seems to be unquestionable; our study supports the urgent need of including LM patients in clinical trials testing novel therapeutic strategies, so to improve the outcomes of this prognostically unfavorable patient subset.

Further collaboration in the presented topic is planned in our international working group BrainMet BC international.

## CRediT authorship contribution statement

**Elena Laakmann:** Writing – original draft, Supervision, Conceptualization. **Marcus Schmidt:** Writing – review & editing. **Kristina Lübbe:** Writing – review & editing. **Elisa Agostinetto:** Writing – review & editing, Conceptualization. **Mette van Ramshorst:** Writing – review & editing, Conceptualization. **Thomas Decker:** Writing – review & editing. **Wolfram Malter:** Writing – review & editing. **Francesco Schettini:** Writing – review & editing, Conceptualization. **Mario Fontes Sousa:** Writing – review & editing, Conceptualization. **Carsten Denkert:** Writing – review & editing. **Tanja Neunhöffer:** Writing – review & editing. **Leonor Matos:** Writing – review & editing, Conceptualization. **Sabine Linn:** Writing – review & editing, Conceptualization. **Marc Thill:** Writing – review & editing. **Rudolf Weide:** Writing – review & editing. **Amanda Fitzpatrick:** Writing – review & editing. **Marta Vaz Batista:** Writing – review & editing. **Christoph Mundhenke:** Writing – review & editing. **Tjoung-Won Park-Simon:** Writing – review & editing. **Fanny Le Du:** Writing – review & editing. **Kerstin Riecke:** Writing – review & editing. **Tanja Fehm:** Writing – review & editing. **Isabel Witzel:** Writing – review & editing. **Julia Rey:** Writing – review & editing, Software, Methodology, Data curation, Conceptualization. **Valentina Nekljudova:** Writing – review & editing, Software, Methodology, Data curation, Conceptualization. **Sibylle Loibl:** Writing – review & editing. **Volkmar Müller:** Writing – review & editing, Conceptualization.

## Availability of data and materials

The data that support the findings of this study are not openly available due to reasons of sensitivity and are available from the corresponding author upon reasonable request.

## Ethics approval and consent to participate

Ethical approval for this study was obtained from Ethikkommission bei der Landesärztekammer Hessen (approval number: FF42/2013).

## Funding

Financial support for the management of BMBC Registry was provided by an unrestricted research grant from Daiichi-Sankyo to GBG. Any views, opinions, findings, conclusions, or recommendations expressed in this material are those solely of the authors and do not necessarily reflect those of funding entities.

## Declaration of competing interest

EA: Honoraria from: Eli Lilly, Sandoz, AstraZeneca, Novartis; Advisory Board for AstraZeneca; Research grant to my Institution from Gilead; Support for attending medical conferences from: Novartis, Roche, Eli Lilly, Genetic, Istituto Gentili, Daiichi Sankyo, AstraZeneca (all outside this manuscript).

LM: reports speaking fee/honoraria from AstraZeneca, Roche, Lilly, Novartis, Daiichi Sankyo; consultancy role for Grunenthal and Novartis; meeting/travel grants from Lilly, Pfizer, Novartis and Daichii Sankyo.

FS: declares personal fees for educational events and/or materials from Gilead, Daiichy Sankyo and Novartis; travel expenses from Gilead, Daiichy Sankyo and Novartis; advisory fees from Pfizer.

JR and VN declares to be GBG Forschungs GmbH employee. GBG Forschungs GmbH received funding for research grants from Abbvie, Amgen, AstraZeneca, BMS, Daiichi-Sankyo, Gilead, Molecular Health, Stemline Menarini, Celgene/BMS, Novartis, Pfizer and Roche (paid to the institution). GBG Forschungs GmbH has licensing fees from VMscope GmbH. In addition, GBG Forschungs GmbH has a patent EP21152186.9 pending, a patent EP19808852.8 pending, and a patent EP14153692.0 pending.

EL reports travel expenses from Pierre Fabre, honoraria for educational events from Astra Zeneca, Seagen and advisory board fees from Novartis, Astra Zeneca and Daiichi Sankyo (all outside this manuscript).

AF, MvR, RW: no relevant disclosures to declare.

MS reports personal fees from AstraZeneca, BioNTech, Daiichi Sankyo, Eisai, Exact Sciences, GILEAD, Lilly, Menarini-Stemline, Molecular Health, MSD, Novartis, Pantarhei Bioscience, Pfizer, Pierre Fabre, Roche, and SeaGen, His institution has received research funding from AstraZeneca, BioNTech, Eisai, Genentech, German Breast Group, Novartis, Palleos, Pantarhei Bioscience, Pierre Fabre, and SeaGen. In addition, he has a patent for EP 2390370 B1 and a patent for EP 2951317 B1 issued.

TF received travel expenses from Daichii Sankyo and Roche, honoraria from Onkowissen, FOMF, Medcocept

IW received honoraria from Astra Zeneca, GSK, Lilly, Novartis, Roche, Seagen, Pfizer, Daiichi Sankyo and Gilead.

MT reports honoraria für advisory board from Agendia, Amgen, AstraZeneca, Aurikamed, Becton/Dickinson, ClearCut, Daiichi Sankyo, Eisai, Exact Sciences, Gilead Science, GSK, Lilly, MSD, Neodynamics, Novartis, Onkowissen, Organon, Pfizer, pfm Medical, Pierre-Fabre, Roche, Seagen, Sirius Medical, Sysmex, manuscript support from Amgen, ClearCut, pfm medical, Roche, Servier, reimbursement of travel expenses from Amgen, Art Tempi, AstraZeneca, Clearcut, Daiichi Sankyo, Eisai, Exact Sciences, Gilead, Hexal, I-Med-Institute, Lilly, MSD, Neodynamics, Novartis, Pfizer, pfm Medical, Roche, RTI Surgical, Seagen, ZP Therapeutics, congress support from Amgen, AstraZeneca, Daiichi Sanyko, Gilead, Hexal, Lilly, Neodynamics, Novartis, Pfizer, Pierre Fabre, Roche, Sirius Medical, moreover honoraria for lectures from Agendia, Amgen, Art Tempi, AstraZeneca, Eisai, Exact Sciences, Gilead Science, GSK, Hexal, I-Med-Institute, Jörg Eickeler, Laborarztpraxis Walther et al., Lilly, Medscape, MSD, Novartis, Onkowissen, Pfizer, pfm medical, Roche, Seagen, StreamedUp, Stemline, Sysmex, Vifor, Viatris, ZP Therapeutics, institutional trial funding from Endomag, Exact Sciences and institutional trial honoraria from AstraZeneca, Biom’Up, CairnSurgical, Clearcut, Neodynamics, Novartis, pfm medical, Roche, RTI Surgical.

FLD: Consultancy fee or honoraria: Eli Lilly, Pfizer, Novartis, Seagen, Daiichi, AstraZeneca, Exact sciences. Research grant to my Institution from Daiichi. Support for attending medical conferences from: Novartis, Roche, Eli Lilly, Pfizer, Daiichi Sankyo, AstraZeneca (all outside the submitted work).

C. Mundhenke reports personal fees from AstraZeneca, Daiichi Sankyo, Eisai, Menarini-Stemline, MSD, Novartis, Pfizer and SeaGen.

VM Speaker honoraria: Astra Zeneca, Daiichi-Sankyo, Eisai, Pfizer, MSD, Medac, Novartis, Roche, Seagen, Onkowissen, high5 Oncology, Medscape, Gilead, Pierre Fabre, iMED Institut.

Consultancy honoraria: Roche, Pierre Fabre, PINK, ClinSol, Novartis, MSD, Daiichi-Sankyo, Eisai, Lilly, Seagen, Gilead, Stemline, Institutional research support: Novartis, Roche, Seagen, Genentech, Astra Zeneca, Travel grants: Astra Zeneca, Roche, Pfizer, Daiichi Sankyo, Gilead. Given his role as specialty editor (medical oncology) of The Breast Journal, Volkmar Mueller had no involvement in the peer-review of this article and has no involvement in the peer review of this article and has no access to information regarding its peer review.

## References

[1] Le Rhun E., Preusser M., Van Den Bent M., Andratschke N., Weller M. (2019). How we treat patients with leptomeningeal metastases. ESMO Open.

[2] Franzoi M.A., Hortobagyi G.N. (2019 Mar). Leptomeningeal carcinomatosis in patients with breast cancer. Crit Rev Oncol Hematol.

[3] Gauthier H., Guilhaume M.N., Bidard F.C., Pierga J.Y., Girre V., Cottu P.H., Laurence V., Livartowski A., Mignot L., Diéras V. (2010). Survival of breast cancer patients with meningeal carcinomatosis. Ann Oncol.

[4] Le Rhun E., Taillibert S., Zairi F. (2013). A retrospective case series of 103 consecutive patients with leptomeningeal metastasis and breast cancer. J Neuro Oncol.

[5] Scott B.J., Oberheim-Bush N.A., Kesari S. (2016). Leptomeningeal metastasis in breast cancer - a systematic review. Oncotarget.

[6] Morikawa A., Jordan L., Rozner R. (2017). Characteristics and outcomes of patients with breast cancer with leptomeningeal metastasis. Clin Breast Cancer.

[7] Müller V., Laakmann E., Fehm T., Möbus V., Von Minckwitz G., Kaiser J., Loibl S., Witzel I. (2015). Brain metastases in breast cancer network Germany (BMBC, GBG 79): multicentric, retrospective and prospective collection of patient data and biomaterial from breast cancer patients as platform for translational research. Ann Oncol.

[8] Riecke K., Müller V., Neunhöffer T., Park-Simon T.W., Weide R., Polasik A., Schmidt M., Puppe J., Mundhenke C., Lübbe K., Hesse T., Thill M., Wuerstlein R., Denkert C., Decker T., Fehm T., Nekljudova V., Rey J., Loibl S., Laakmann E., Witzel I. (2023). Long-term survival of breast cancer patients with brain metastases: subanalysis of the BMBC registry. ESMO Open.

[9] Laakmann E., Witzel I., Neunhöffer T., Park-Simon T.W., Weide R., Riecke K., Polasik A., Schmidt M., Puppe J., Mundhenke C., Lübbe K., Hesse T., Thill M., Zahm D.M., Denkert C., Fehm T., Nekljudova V., Rey J., Loibl S., Müller V. (2022 Jun). Characteristics of patients with brain metastases from human epidermal growth factor receptor 2-positive breast cancer: subanalysis of Brain Metastases in Breast Cancer Registry. ESMO Open.

[10] Laakmann E., Witzel I., Neunhöffer T., Park-Simon T.-W., Weide R., Riecke K., Polasik A., Schmidt M., Puppe J., Mundhenke C., Lübbe K., Hesse T., Thill M., Zahm D.M., Denkert C., Fehm T., Nekljudova V., Rey J., Loibl S., Müller V. (2022). Characteristics of patients with brain metastases from human epidermal growth factor receptor 2-positive breast cancer: subanalysis of brain metastases in breast cancer registry. ESMO Open.

[11] Le Rhun E., Weller M., van den Bent M., on behalf of the EANO Guidelines Committee and ESMO Guidelines Committee (2023). Leptomeningeal metastasis from solid tumours: EANO–ESMO Clinical Practice Guideline for diagnosis, treatment and follow-up. ESMO Open.

[12] Allison K.H., Hammond M.E.H., Dowsett M. (2020). Estrogen and progesterone receptor testing in breast cancer: ASCO/CAP guideline update. J Clin Orthod.

[13] Wolff A.C., Hammond M.E.H., Allison K.H. (2018). Human epidermal growth factor receptor 2 testing in breast cancer: American Society of Clinical Oncology/College of American Pathologists Clinical Practice guideline focused update. J Clin Oncol.

[14] Abouharb S., Ensor J., Loghin M.E., Katz R., Moulder S.L., Esteva F.J., Smith B., Valero V., Hortobagyi G.N., Melhem-Bertrandt A. (2014). Leptomeningeal disease and breast cancer: the importance of tumor subtype. Breast Cancer Res Treat.

[15] Griguolo G., Pouderoux S., Dieci M.V., Jacot Bourgier C., Miglietta F., Firmin N., Conte P., Viala M., Guarneri V., Darlix A. (2018). Clinicopathological and treatment-associated prognostic factors in patients with breast cancer leptomeningeal metastases in relation to tumor biology. Oncologist.

[16] Niwińska A., Rudnicka H., Murawska M. (2013). Breast cancer leptomeningeal metastasis: propensity of breast cancer subtypes for leptomeninges and the analysis of factors influencing survival. Med Oncol.

[17] Torrejón D., Oliveira M., Cortes J., Sanchez-Olle G., Gómez P., Bellet M., Saura C., Peg V., Rovira A., Di Cosimo S. (2013). Implication of breast cancer phenotype for patients with leptomeningeal carcinomatosis. Breast.

[18] Fitzpatrick A., Iravani M., Mills A., Vicente D., Alaguthurai T., Roxanis I., Turner N.C., Haider S., Tutt A.N.J., Isacke C.M. (2023 Nov 16). Genomic profiling and pre-clinical modelling of breast cancer leptomeningeal metastasis reveals acquisition of a lobular-like phenotype. Nat Commun.

[19] De Azevedo C.R.A.S., Cruz M.R.S., Chinen L.T.D., Peres S.V., Peterlevitz M.A., De Azevedo Pereira A.E., Fanelli M.F., Gimenes D.L. (2011). Meningeal carcinomatosis in breast cancer: prognostic factors and outcome. J Neuro Oncol.

[20] Hyun J.W., Jeong I.H., Joung A., Cho H.J., Kim S.H., Kim H.J. (2016). Leptomeningeal metastasis: clinical experience of 519 cases. Eur J Cancer.

[21] Witzel I., Laakmann E., Weide R., Neunhöffer T., Park-Simon T.J., Schmidt M., Fasching P.A., Hesse T., Polasik A., Mohrmann S., Würschmidt F., Schem C., Bechtner C., Würstlein R., Fehm T., Möbus V., Burchardi N., Loibl S., Müller V. (2018). Treatment and outcomes of patients in the brain metastases in breast cancer network registry. Eur J Cancer.

[22] Bartsch R., Berghoff A.S., Furtner J., Marhold M., Bergen E.S., Roider-Schur S., Starzer A.M., Forstner H., Rottenmanner B., Dieckmann K., Bago-Horvath Z., Haslacher H., Widhalm G., Ilhan-Mutlu A., Minichsdorfer C., Fuereder T., Szekeres T., Oehler L., Gruenberger B., Singer C.F., Weltermann A., Puhr R., Preusser M. (2022 Sep). Trastuzumab deruxtecan in HER2-positive breast cancer with brain metastases: a single-arm, phase 2 trial. Nat Med.

[23] Lin N.U., Murthy R.K., Abramson V., Anders C., Bachelot T., Bedard P.L., Borges V., Cameron D., Carey L.A., Chien A.J., Curigliano G., DiGiovanna M.P., Gelmon K., Hortobagyi G., Hurvitz S.A., Krop I., Loi S., Loibl S., Mueller V., Oliveira M., Paplomata E., Pegram M., Slamon D., Zelnak A., Ramos J., Feng W., Winer E. (2023 Feb 1). Tucatinib vs placebo, both in combination with trastuzumab and capecitabine, for previously treated ERBB2 (HER2)-Positive metastatic breast cancer in patients with brain metastases: updated exploratory analysis of the HER2CLIMB randomized clinical trial. JAMA Oncol.

[24] Carey L.A., Loirat D., Punie K., Bardia A., Diéras V., Dalenc F., Diamond J.R., Fontaine C., Wang G., Rugo H.S., Hurvitz S.A., Kalinsky K., O'Shaughnessy J., Loibl S., Gianni L., Piccart M., Zhu Y., Delaney R., Phan S., Cortés J. (2022 Jun 9). Sacituzumab govitecan as second-line treatment for metastatic triple-negative breast cancer-phase 3 ASCENT study subanalysis. NPJ Breast Cancer.

[25] Fernandes L., de Matos L.V., Cardoso D., Saraiva M., Medeiros-Mirra R., Coelho A., Miranda H., Martins A. (2020 Dec 1). Endocrine therapy for the treatment of leptomeningeal carcinomatosis in luminal breast cancer: a comprehensive review. CNS Oncol.

[26] Le Rhun E., Wallet J., Mailliez A., Le Deley M.C., Rodrigues I., Boulanger T., Lorgis V., Barrière J., Robin Y.M., Weller M., Bonneterre J. (2020 Apr 15). Intrathecal liposomal cytarabine plus systemic therapy versus systemic chemotherapy alone for newly diagnosed leptomeningeal metastasis from breast cancer. Neuro Oncol.

[27] Marowsky M., Müller V., Schmalfeldt B., Riecke K., Witzel I., Laakmann E. (2023 Nov 8). Intrathecal therapy options for meningeal carcinomatosis. Geburtshilfe Frauenheilkd.

