# Supplementary material for: Clinical characteristics and prognostic factors in patients with breast cancer and leptomeningeal metastases from a large registry of BMBC
Source: Breast. 2025 Mar 28;81:104433. doi: 10.1016/j.breast.2025.104433 (PMC12002880; doi:10.1016/j.breast.2025.104433)
Supplement: Multimedia component 1 [file mmc1.docx]

**Supplementary material**

**Supplementary material Table 1:**

| Parameter | Category | *Patients without LM*  *N=2998 N (%)* | *Patients with LM*  *N=859 N (%)* | *Overall N=3857 N (%)* | P-Value |
| --- | --- | --- | --- | --- | --- |
| Bone metastases as first ECM | no | 1859 (62.0) | 418 (48.7) | 2277 (59.1) | <.001 |
|  | yes | 1138 (38.0) | 441 (51.3) | 1579 (40.9) |  |
|  | missing | 1 | 0 | 1 |  |
| Liver metastases as first ECM | no | 2200 (73.4) | 686 (79.9) | 2886 (74.8) | <.001 |
|  | yes | 797 (26.6) | 173 (20.1) | 970 (25.2) |  |
|  | missing | 1 | 0 | 1 |  |
| Lung metastases as first ECM | no | 1969 (65.7) | 686 (79.9) | 2655 (68.9) | <.001 |
|  | yes | 1028 (34.3) | 173 (20.1) | 1201 (31.1) |  |
|  | missing | 1 | 0 | 1 |  |
| Skin metastases as first ECM | no | 2856 (95.3) | 825 (96.0) | 3681 (95.5) | 0.403 |
|  | yes | 141 ( 4.7) | 34 ( 4.0) | 175 ( 4.5) |  |
|  | missing | 1 | 0 | 1 |  |
| Other metastases as first ECM | no | 2170 (72.4) | 643 (74.9) | 2813 (73.0) | 0.163 |
|  | yes | 827 (27.6) | 216 (25.1) | 1043 (27.0) |  |
|  | missing | 1 | 0 | 1 |  |

Legend: LM leptomeningeal metastases, ECM extracranial metastases

**Supplementary Table 2. Local surgical and radiotherapy of CNS metastases**

| *Parameter* | *Category* | *Patients without LM N=2998 N(%)* | *Patients with LM N=859 N(%)* | *Overall N=3857 N(%)* | *p-value** |
| --- | --- | --- | --- | --- | --- |
| Local treatment of BM | Surgery only | 156 ( 6.1) | 38 ( 6.2) | 194 ( 6.1) | 0.055 |
|  | RTH only | 1736 (67.3) | 440 (71.9) | 2176 (68.2) |  |
|  | Surgery and RTH | 686 (26.6) | 134 (21.9) | 820 (25.7) |  |
|  | missing | 420 | 247 | 667 |  |
| Type of Radiotherapy | WBRT only | 1805 (60.2) | 467 (54.4) | 2272 (58.9) | <.001 |
|  | Stereo RT only | 393 (13.1) | 79 ( 9.2) | 472 (12.2) |  |
|  | WBRT and Stereo RT | 163 ( 5.4) | 17 ( 2.0) | 180 ( 4.7) |  |
|  | RTH unknown | 61 ( 2.0) | 11 ( 1.3) | 72 ( 1.9) |  |
|  | No RTH | 575 (19.2) | 284 (33.1) | 859 (22.3) |  |
|  | missing | 1 | 1 | 2 |  |

**Supplementary Table 3: Univariate Cox Regression of the time from BM to death in patients with LM**

| *Parameter* | *Category* | *Number of events* | *Hazard Ratio* | *SE* | *95%-CI* | *p-value* |
| --- | --- | --- | --- | --- | --- | --- |
| Age at diagnosis of BC |  |  |  |  |  |  |
|  | <60 | 509 (89.8) |  |  |  |  |
|  | ≥60 | 218 (92.8) | 1.44 | .081 | (1.22, 1.68) | <.001 |
| Age at diagnosis of BM |  |  |  |  |  |  |
|  | <60 | 396 (89.0) |  |  |  |  |
|  | ≥60 | 331 (92.7) | 1.45 | .075 | (1.25, 1.68) | <.001 |
| Biological subtypes* |  |  |  |  |  | <.001 |
|  | HR+/HER2+ | 140 (85.4) |  | . |  |  |
|  | HR-/HER2+ | 69 (79.3) | 1.05 | .148 | (.786, 1.40) | 0.737 |
|  | HR+/HER2- | 299 (92.9) | 1.63 | .103 | (1.33, 2.00) | <.001 |
|  | TNBC | 154 (95.7) | 2.21 | .119 | (1.75, 2.79) | <.001 |
| Number of BM |  |  |  |  |  | <.001 |
|  | 1 | 205 (86.9) |  | . |  |  |
|  | 2-3 | 136 (91.3) | 1.35 | .112 | (1.08, 1.68) | 0.008 |
|  | ≥4 | 213 (91.0) | 1.47 | .099 | (1.21, 1.78) | <.001 |
| HR status |  |  |  |  |  |  |
|  | both ER and PgR negative | 234 (90.0) |  |  |  |  |
|  | ER and/or PgR positive | 463 (90.6) | .835 | .081 | (.713, .979) | .026 |
| HER2 status at diagnosis of BC |  |  |  |  |  |  |
|  | negative | 454 (93.8) |  |  |  |  |
|  | positive | 202 (83.5) | .563 | .086 | (.476, .667) | <.001 |
| ECOG |  |  |  |  |  |  |
|  | ECOG 0-1 | 173 (84.8) |  |  |  |  |
|  | ECOG 2-4 | 170 (95.0) | 2.23 | .111 | (1.79, 2.77) | <.001 |
| Clinical symptoms |  |  |  |  |  |  |
|  | no | 123 (86.0) |  |  |  |  |
|  | yes | 605 (91.7) | 1.52 | .099 | (1.25, 1.85) | <.001 |
| ECM at BM diagnosis |  |  |  |  |  |  |
|  | no | 136 (85.5) |  |  |  |  |
|  | yes | 592 (91.9) | 1.32 | .095 | (1.10, 1.60) | 0.003 |
| Radiotherapy |  |  |  |  |  | <.001 |
|  | No RT | 250 (94.0) |  | . |  |  |
|  | WBRT only | 397 (90.8) | .605 | .081 | (.516, .709) | <.001 |
|  | Stereo RT only | 58 (76.3) | .378 | .146 | (.284, .504) | <.001 |
|  | WBRT and Stereo RT | 12 (92.3) | .379 | .296 | (.212, .677) | 0.001 |
|  | RT unknown | 10 ( 100) | .560 | .323 | (.297, 1.05) | 0.072 |
| Chemotherapy after diagnosis of BM |  |  |  |  |  |  |
|  | no | 438 (91.3) |  |  |  |  |
|  | yes | 290 (89.8) | .548 | .076 | (.471, .636) | <.001 |
| Hormone therapy after diagnosis of BM |  |  |  |  |  |  |
|  | no | 624 (92.6) |  |  |  |  |
|  | yes | 104 (80.6) | .409 | .108 | (.332, .506) | <.001 |
| HER2-targeted therapy after diagnosis of BM |  |  |  |  |  |  |
|  | no | 648 (92.3) |  |  |  |  |
|  | yes | 80 (79.2) | .392 | .121 | (.310, .497) | <.001 |
| Intrathecal therapy after diagnosis of BM |  |  |  |  |  |  |
|  | no | 673 (90.1) |  |  |  |  |
|  | yes | 55 (98.2) | .908 | .140 | (.689, 1.20) | 0.492 |

**Legend.** BM: brain metastasis; FU: follow-up; WBRT: whole breast radiotherapy; RT: radiotherapy; HR: hormone receptor; +: positive; -: negative; ECM: extracranial metastases; ER: estrogen receptor; PgR: progesterone receptor; BC: breast cancer.

* If HER2-Status at diagnosis of BC was unknown, but Anti-HER2-targeted therapy was given, the subtype was set to HR+/HER2+ resp. HR-/HER2+ (if information about HR-status was given, too)

**Supplementary Figure 1: Overall survival of patients with leptomeningeal disease, per subtype**


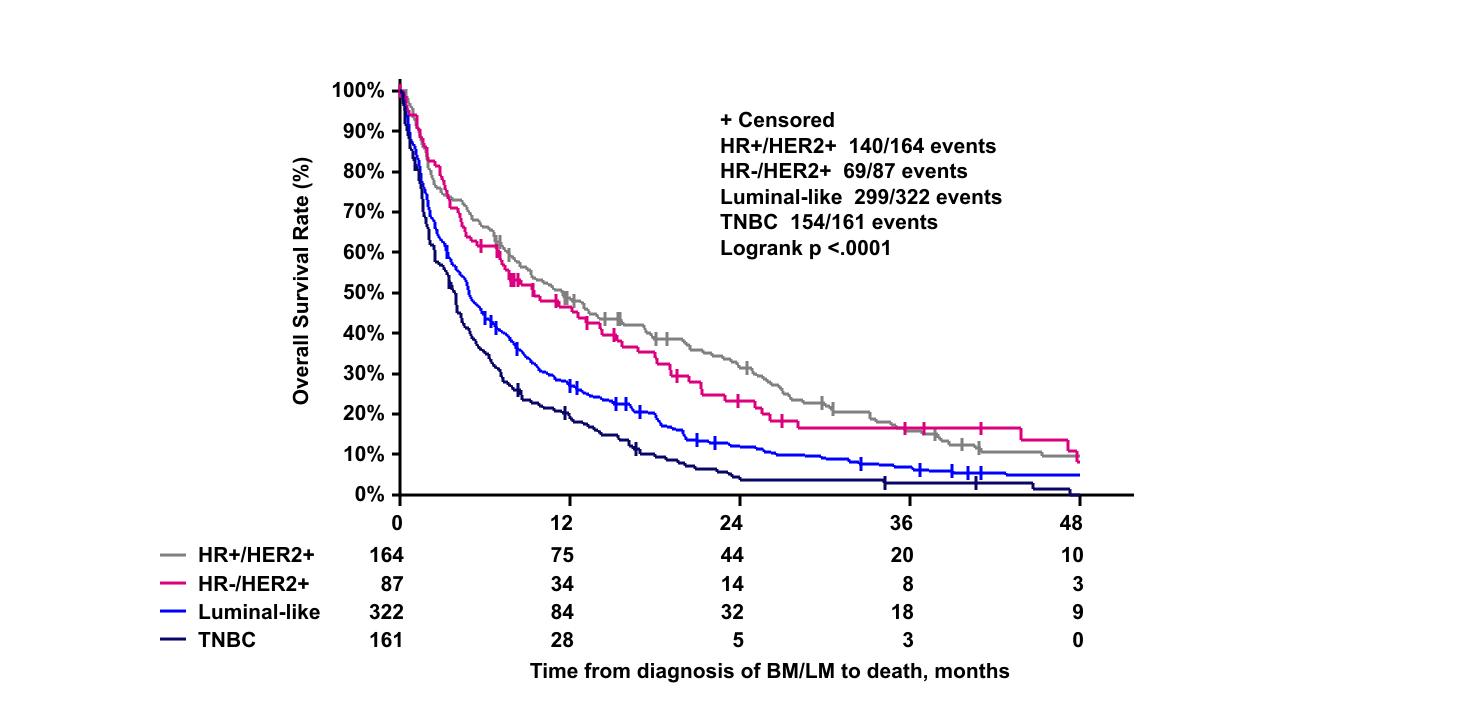


| *Time* | *HR+/HER2+* | *HR-/HER2+* | *Luminal A/B like* | *TNBC* |
| --- | --- | --- | --- | --- |
| Estimated 1 year OS (95% CI) | 48.7% (40.8%, 56.1%) | 46.6% (35.6%, 56.8%) | 27.1% (22.4%, 32.1%) | 18.8% (13.1%, 25.3%) |
| Estimated 2 year OS (95% CI) | 31.5% (24.2%, 38.9%) | 23.2% (14.2%, 33.4%) | 11.7% ( 8.4%, 15.7%) | 3.6% ( 1.4%, 7.5%) |
| Estimated 3 year OS (95% CI) | 15.7% (10.2%, 22.2%) | 16.4% ( 8.7%, 26.2%) | 6.9% ( 4.4%, 10.3%) | 2.9% ( 1.0%, 6.6%) |
| Estimated 4 year OS (95% CI) | 9.6% ( 5.3%, 15.5%) | 8.2% ( 2.5%, 18.3%) | 4.8% ( 2.6%, 7.8%) | 1.4% ( 0.2%, 5.7%) |

**Supplementary Figure 2 Kaplan-Meier curves for the time from diagnosis of BM to death between patients with vs. without cytologically confirmed leptomeningeal disease**

| **OS rates for patients with vs. without cytologically confirmed meningiosis in patients with leptomeningeal metastases** |
| --- |

| *Time* | *Pts without cerebrospinal fluid analysis* | *Pts with positive cerebrospinal fluid* |
| --- | --- | --- |
| Estimated 1 year OS (95% CI) | 34.9% (30.3%, 39.5%) | 30.0% (25.4%, 34.8%) |
| Estimated 2 year OS (95% CI) | 15.6% (12.1%, 19.4%) | 17.0% (13.3%, 21.2%) |
| Estimated 3 year OS (95% CI) | 9.6% ( 6.8%, 12.9%) | 8.1% ( 5.5%, 11.4%) |
| Estimated 4 year OS (95% CI) | 5.7% ( 3.5%, 8.8%) | 5.4% ( 3.2%, 8.4%) |

**Legend.** Pts: patients; OS: overall survival; CI: confidence interval.
